# Maximizing Nano-Silica Efficiency in Laboratory-Simulated Recycled Concrete Aggregate via Prior Accelerated Carbonation: An Effective Strategy to Up-Cycle Construction Wastes

**DOI:** 10.3390/molecules29245995

**Published:** 2024-12-19

**Authors:** Cheng-Gong Lu, Xiu-Cheng Zhang, Xue-Fei Chen

**Affiliations:** 1 School of Civil Engineering and Transportation, Guangzhou University, Guangzhou 510006, China; 2 School of Civil Engineering, Putian University, Putian 351100, China; 3 Engineering Research Center of Disaster Prevention and Mitigation of Southeast Coastal Engineering Structures (JDGC03), Fujian Province University, Putian 351100, China

**Keywords:** construction wastes, recycled aggregates, concrete

## Abstract

Herein, the study explores a composite modification approach to enhance the use of recycled concrete aggregate (RCA) in sustainable construction by combining accelerated carbonation (AC) and nano-silica immersion (NS). RCA, a major source of construction waste, faces challenges in achieving comparable properties to virgin aggregates. Nano-silica, a potent pozzolan, is added to fill micro-cracks and voids in RCA, improving its bonding and strength. AC pretreatment accelerates RCA’s natural carbonation, forming calcium carbonate that strengthens the aggregate and reduces porosity. Due to the complexity of the original RCA, a laboratory-simulated RCA (LS-RCA) is used in this study for the mechanism analysis. Experimental trials employing the composite methodology have exhibited noteworthy enhancements, with the crushing index diminishing by approximately 23% and water absorption rates decreasing by up to 30%. Notably, the modification efficacy is more pronounced when applied to RCA derived from common-strength concrete (w/c of 0.5) as compared to high-strength concrete (w/c of 0.35). This disparity stems from the inherently looser structural framework and greater abundance of detrimental crystal structures in the former, which impede strength. Through a synergistic interaction, the calcium carbonate content undergoes a substantial increase, nearly doubling, while the proportion of calcium hydrate undergoes a concurrent reduction of approximately 30%. Furthermore, the combined modification effect leads to a 15% reduction in total porosity and a constriction of the average pore diameter by roughly 20%, ultimately resulting in pore refinement that equates the performance of samples with a water-to-cement ratio of 0.5 to those with a ratio of 0.35. This remarkable transformation underscores the profound modification potential of the combination approach. This study underscores the efficacy of harnessing accelerated carbonation in conjunction with nano-silica as a strategic approach to optimizing the utilization of RCA in concrete mixes, thereby bolstering their performance metrics and enhancing sustainability.

## 1. Introduction

In the context of rapid urbanization and unprecedented infrastructure development globally, the construction industry has emerged as a pivotal driver of economic growth [1]. However, this progressive trajectory is marred by a pressing environmental challenge: the massive generation of construction and demolition waste (CDW) [2,3]. Statistics reveal that the construction sector accounts for a substantial portion of global solid waste, with estimates suggesting that the annual production of CDW could exceed 2 billion tons worldwide [4]. The traditional approach to managing this deluge of waste has largely revolved around landfilling, an unsustainable practice that not only consumes vast amounts of land resources but also poses long-term threats to groundwater quality and ecological balance [5]. As the world grapples with the realities of climate change and resource depletion, transitioning from a linear ‘take-make-dispose’ model to a circular economy has become imperative.

One promising solution to this dilemma lies in the conversion of CDW into recycled aggregates (RAs) for reuse in construction materials. The adoption of RAs as a substitute for natural aggregates (NAs) in concrete production represents a significant step towards achieving sustainability in the construction sector [6,7]. It not only alleviates the pressure on natural resources by reducing the demand for quarrying, but also mitigates the environmental impacts associated with CDW disposal. Moreover, the reuse of RAs contributes to reducing the carbon footprint of the construction industry by avoiding the energy-intensive processes involved in the extraction and processing of virgin aggregates [8].

Despite these advantages, the widespread adoption of RAs in concrete construction faces several obstacles, primarily stemming from their inherent properties [9]. RAs, being composed of aged mortar attached to the original aggregate particles and often containing internal microcracks, tend to exhibit inferior physical and mechanical properties compared to NAs [10,11]. These deficiencies, such as lower density, increased water absorption, and reduced strength, can negatively impact the performance of concrete made with RAs, limiting their application in high-strength or structural concrete [12,13]. Therefore, the development of effective methods to enhance the properties of RAs has become a crucial research focus.

Over the years, researchers have explored various strategies to improve the performance of RAs. One common approach involves mechanical treatment, such as crushing and grinding [14], which aims to remove the weakly bonded mortar matrix and refine the particle size distribution. While this method can partially address the issue of higher water absorption, it may also lead to the generation of new microcracks and an increase in the surface area of the RAs, potentially exacerbating their performance deficiencies [15]. Another category of enhancement techniques involves chemical treatment, which seeks to modify the surface properties of RAs. Among these, thermal treatment [16] has been shown to reduce water absorption and increase the strength of RAs by promoting the dehydration of the mortar matrix. However, this method is energy-intensive and may not be economically viable on a large scale.

In recent years, the use of nanoparticles, particularly nano-silica (nSiO_2_), has garnered significant attention as a highly effective means of enhancing the properties of RAs [17,18,19]. Nano-silica, known for its exceptional surface area and reactivity, can form a dense, siliceous layer on the surface of RAs upon interaction, thereby improving their mechanical strength, reducing water absorption, and enhancing durability [17]. Several studies [20,21,22] have demonstrated the effectiveness of nSiO_2_ in improving the performance of RAs and the concrete made with them through various application methods, including direct mixing, surface coating, and immersion. The immersion method in particular has been widely studied due to its simplicity and potential for industrial-scale application. In this approach, RAs are submerged in a solution containing nSiO_2_ for a prolonged period, allowing the nanoparticles to penetrate the pores and microcracks of the aggregate surface and form a reinforcing layer [20]. When RAs are immersed in a nSiO_2_ solution, the nanoparticles penetrate into the pores and microcracks, reacting with the calcium hydroxide (CH) and other hydrated calcium silicates present in the aggregate matrix to form calcium silicate hydrate (C-S-H) gels. This reaction not only densifies the pore structure but also enhances the interlocking between the old and new mortar, leading to improved mechanical properties and durability [23].

While this method has shown promising results in terms of improving the physical and mechanical properties of RAs, it is not without drawbacks. One of the primary limitations of the traditional immersion method is the inefficient utilization of nSiO_2_ [22]. Given the high cost of nanomaterials, it is imperative to maximize their effectiveness during the treatment process. The traditional immersion method often results in a significant portion of the nanoparticles being wasted due to incomplete absorption by the aggregate surface. Furthermore, the excessive accumulation of nano-SiO_2_ within the aggregate pores can lead to clogging, which may adversely affect the hydration process and the overall performance of the resulting concrete [21,23].

In response to these challenges, accelerated carbonation emerges as a promising alternative method for enhancing the properties of RAs [24]. This process leverages the natural phenomenon of carbonation, wherein carbon dioxide (CO_2_) reacts with calcium-bearing minerals in the presence of moisture to form CaCO_3_. By exposing RAs to a controlled environment with an elevated concentration of CO_2_, the accelerated carbonation process can be significantly accelerated, leading to a more pronounced modification of the aggregate’s microstructure [25]. The advantages of accelerated carbonation are multifaceted. Firstly, it offers an environmentally friendly solution by sequestering CO_2_, thereby contributing to the mitigation of climate change. In the context of construction waste management, this process effectively transforms a greenhouse gas into a beneficial material modifier. Secondly, accelerated carbonation acts as a natural densifier, reducing the porosity and permeability of RAs by filling pores and microcracks with CaCO_3_. This refinement of the pore structure enhances the mechanical strength and durability of the aggregates [26].

Crucially, accelerated carbonation also presents an opportunity to optimize the utilization of nSiO_2_ in the enhancement of LS-RAs. By preconditioning LS-RAs with an accelerated carbonation treatment, the resulting finer pore structure and reduced porosity [27] may improve the absorption efficiency of nSiO_2_ during subsequent immersion. The combination of accelerated carbonation and nSiO_2_ immersion could therefore lead to a more effective and efficient reinforcement process, maximizing the benefits of both methods while minimizing waste. The combination of accelerated carbonation and nSiO_2_ immersion holds significant potential for the development of high-performance LS-RAs. However, the precise mechanisms underpinning this synergy, as well as the optimal conditions for achieving maximum enhancement, remain largely unexplored. The core research inquiries in this study encompass a multifaceted examination aimed at elucidating the intricate mechanisms underpinning the modification of pore structure and mineralogy in LS-RAs through the accelerated carbonation processes. Specifically, we seek to understand how these alterations impact the subsequent absorption capacity of nano-silica (nSiO_2_), thereby revealing the underlying microstructural evolution that governs this phenomenon. The overarching goal of this research endeavor is to disclose the intricate interplay between accelerated carbonation, pore architecture, mineral composition, and the sorption of nSiO_2_ within LS-RAs, offering valuable insights into the optimization of material properties for sustainable construction applications.

## 2. Results and Discussion

### 2.1. Crushing Index and Water Absorption

**Figure 1:**
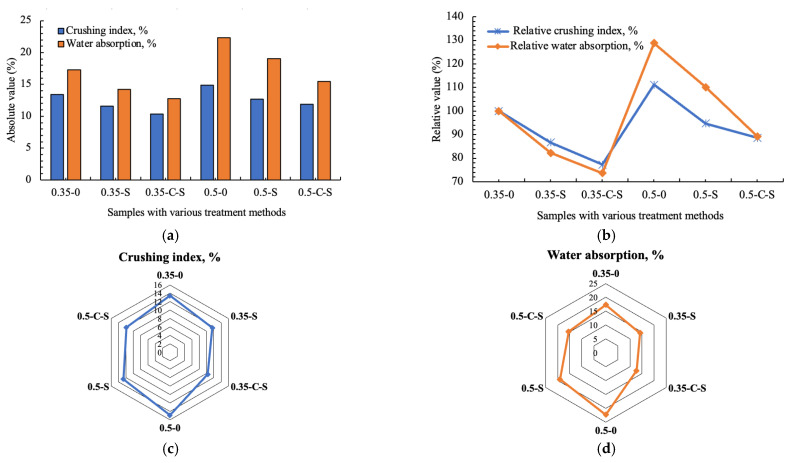
Crushing index and water absorption of samples prepared by various treatment methods: (**a**) the absolute crushing index and water absorption; (**b**) the relative crushing index and water absorption [set the original sample with 0.35 w/b as the benchmark]; (**c**) the radar map of crushing index; (**d**) the radar map of the water absorption.

Figure 1 comprehensively illustrates the comparative analysis of the crushing index and water absorption capabilities of specimens subjected to diverse treatment methodologies. Notably, when focusing on samples formulated with a water-to-binder (w/b) ratio of 0.35, the solitary application of nano-silica treatment yields a substantial decrease in the crushing index of 13.4%, highlighting its potential to enhance mechanical durability. In contrast, the synergistic effect of accelerated carbonation coupled with nano-silica modification yields an even more pronounced reduction of 22.8% in the crushing index, surpassing the standalone nano-silica treatment by approximately 10%, thus underscoring the added benefits of this combined approach.

Turning attention to water absorption, a crucial indicator of material permeability, the introduction of nano-silica modification alone results in a noteworthy decline of 17.8%, demonstrating its effectiveness in reducing porosity and enhancing resistance to moisture ingress. Remarkably, the combined strategy of accelerated carbonation and nano-silica treatment further amplifies this effect, achieving a 26.4% reduction in water absorption, which is approximately 1.5 times more effective than nano-silica treatment alone. This underscores the complementary nature of these two techniques in enhancing the overall impermeability of the samples.

Furthermore, when examining samples with a higher w/b ratio of 0.5, the strengthening effect observed in terms of the crushing index remains comparable to that of samples with a w/b ratio of 0.35, suggesting that the improvements in mechanical properties are not significantly impacted by the change in the w/b ratio. However, a more pronounced enhancement in permeability resistance, as evidenced by a more significant reduction in water absorption, is observed for samples with a w/b ratio of 0.5. Specifically, the combined accelerated carbonation and nano-silica immersion treatment leads to a 30% decline in water absorption for these samples, representing a remarkable improvement of 16.8% over the samples with a w/b ratio of 0.35. This finding underscores the potential of the combined treatment to address the inherent challenges associated with higher w/b ratios, particularly in terms of mitigating the negative effects of increased porosity and enhancing the overall durability of the material.

In conclusion, the present macro-level results demonstrate that while nano-silica modification alone is effective in improving both mechanical properties and permeability resistance, the combined application of accelerated carbonation and nano-silica treatment yields even more substantial enhancements, particularly in terms of reducing water absorption and further strengthening the material. These findings have important implications for the development of high-performance construction materials with enhanced durability and resistance to environmental degradation.

### 2.2. TG Analysis

**Figure 2:**
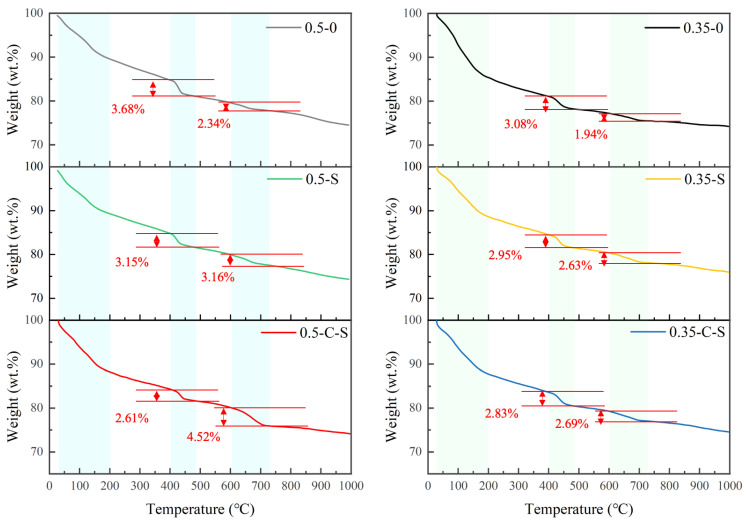
TG curves and calculated content of hydration phases based on the mass losses during thermal decomposition.

**Figure 3:**
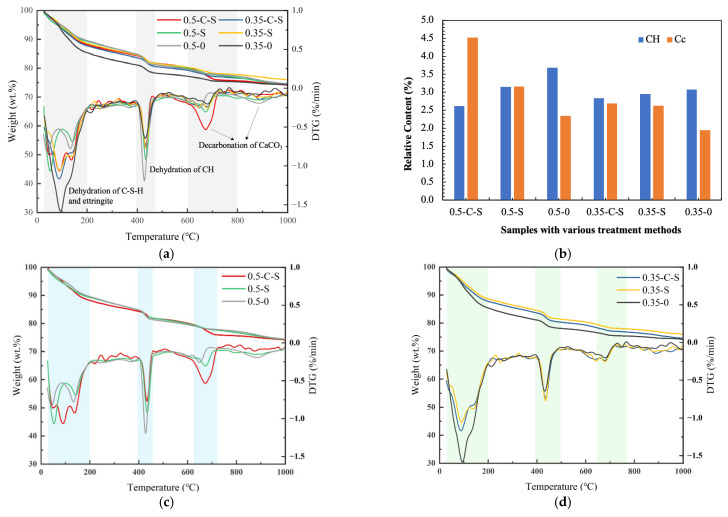
Experimental and calculated TG-DTG curves and their deviations for samples with various treatment methods: (**a**) the overall curves; (**b**) the mass loss of CH and calcium carbonate (Cc); (**c**) the samples with w/b = 0.5; (**d**) the samples with w/b = 0.35.

Figure 2 and Figure 3 exhibit the comprehensive thermal gravimetric analysis (TGA) and derivative thermogravimetry (DTG) curves pertaining to samples subjected to diverse treatment methodologies. These curves reveal three prominent peaks, each corresponding to distinct thermal decomposition events: the initial loss of free water, the subsequent dehydration of calcium silicate hydrate (C-S-H) and ettringite phases, the dehydration of calcium hydroxide (CH), and finally, the de-carbonization of calcium carbonate (Cc). Given that CH and Cc are crystalline, their associated weight losses can serve as quantitative indicators of their relative abundances within the treated mixtures. The TG curves depicted in Figure 2 determine the mass losses associated with hydration phases, primarily CH and Cc, during thermal decomposition. Specifically, the decomposition temperature range for CH is identified as 400–490 °C, while that for Cc is 600–730 °C, based on the peaks observed in the DTG curves presented in Figure 3.

Based on the experimental results, it is evident that samples treated with nano-silica at a water-to-binder ratio (w/b) of 0.5 exhibit an 85.6% retention of CH content in comparison to the control samples (i.e., those untreated ones). This observation underscores the effectiveness of nano-silica in lowering the CH content, despite the inherent changes introduced by the treatment process. Furthermore, when accelerated carbonation is employed as a precursor step prior to nano-silica immersion, the CH content is further reduced to approximately 70.9% of the control value, marking a decrease of approximately 15% from the nano-silica-only treatment. This pronounced reduction in CH content, coupled with the structural modifications induced by both treatments, rationalizes the enhancement in compressive strength, as manifested by the improved crushing index discussed in the preceding section.

Regarding the Cc content, the combined effect of accelerated carbonation followed by nano-silica treatment is particularly noteworthy. This combined approach nearly doubles the Cc content compared to untreated samples, emphasizing its pivotal role in enhancing the overall hardness and durability of the treated aggregates. This augmentation in Cc content can be attributed to the accelerated carbonation process, which favors the formation of Cc, and the subsequent nano-silica treatment, which may further stabilize the newly formed calcite phase.

However, when examining samples prepared at a lower water-to-cement ratio (w/c) of 0.35, the changes in weight associated with both CH and Cc are comparatively negligible, with variations ranging between 5% and 8%. This observation highlights the influence of the initial mixture composition on the treatment outcomes, suggesting that the effectiveness of nano-silica and accelerated carbonation treatments may be more pronounced in samples with higher water content or specific binder-to-water ratios. Overall, the TG analysis provides valuable insights into the intricate interplay between treatment methodologies, mixture composition, and the resultant microstructural changes, which ultimately dictate the mechanical properties of treated aggregates

### 2.3. Porosity Analysis

**Figure 4:**
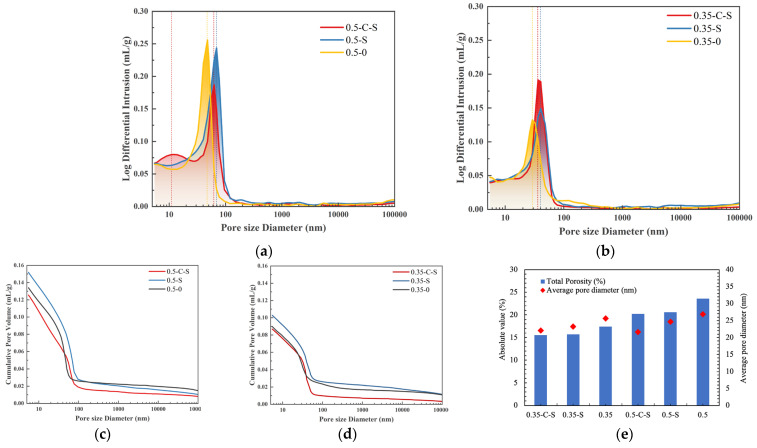
Porosity analysis: (**a**) pore size distribution of samples with w/c = 0.5; (**b**) pore size distribution of samples with w/c = 0.35; (**c**) total porosity of samples with w/c = 0.5; (**d**) total porosity of samples with w/c = 0.35; (**e**) total porosity and the average pore diameter.

Figure 4 comprehensively showcases the porosity outcomes derived from the Mercury Intrusion Porosimetry (MIP) testing procedure, offering valuable insights into the microstructural characteristics of the analyzed samples. Notably, a discernible trend emerges when comparing specimens featuring water-to-cement (w/c) ratios of 0.5 and 0.35. Specifically, the specimens with a higher w/c ratio of 0.5 exhibit a notably elevated total porosity, along with augmented most probable pore diameter and average pore diameter, relative to their counterparts with a w/c ratio of 0.35. This observation underscores the influence of w/c ratio on porosity development and pore size distribution within cementitious materials.

Quantitatively, the total porosity of samples with a w/c ratio of 0.5 surpasses that of the 0.35 w/c samples by a substantial margin of approximately 35%. This finding underscores the direct correlation between increased water content during mixing and the resultant porosity enhancement, likely attributed to the formation of additional pores during hydration and consolidation [28]. However, the narrative shifts significantly when the samples undergo a combined treatment regimen involving accelerated carbonation and nano-silica soaking. This innovative approach yields intriguing results, as the porosity differential between the 0.5 w/c and 0.35 w/c samples narrows considerably. Specifically, the total porosity of the 0.5 w/c samples, post-treatment, remains merely 10% higher than that of the 0.35 w/c samples, indicative of a pronounced mitigation effect.

Furthermore, the analysis of average pore size reveals an unexpected reversal in the trend. Following the combined treatment, the average pore size of the 0.5 w/c samples was found to be significantly lower (by approximately 80%) compared to the 0.35 w/c samples. This remarkable outcome suggests that the synergistic interaction between accelerated carbonation and nano-silica immersion not only reduces overall porosity but also refines the pore structure, favoring the formation of finer pores. Lastly, with respect to the most probable pore diameter, both the 0.5 w/c and 0.35 w/c samples exhibit a notable narrowing effect, with reductions of 20% and 14%, respectively. This observation underscores the efficacy of the combined treatment in refining the pore diameter distribution, further corroborating its ability to modify the microstructure in a beneficial manner. In conclusion, the porosity analysis highlights the complex interplay between w/c ratio, accelerated carbonation, nano-silica immersion, and the resultant microstructural evolution, offering valuable insights for the optimization of RCA.

### 2.4. Strengthening Mechanism

**Figure 5:**
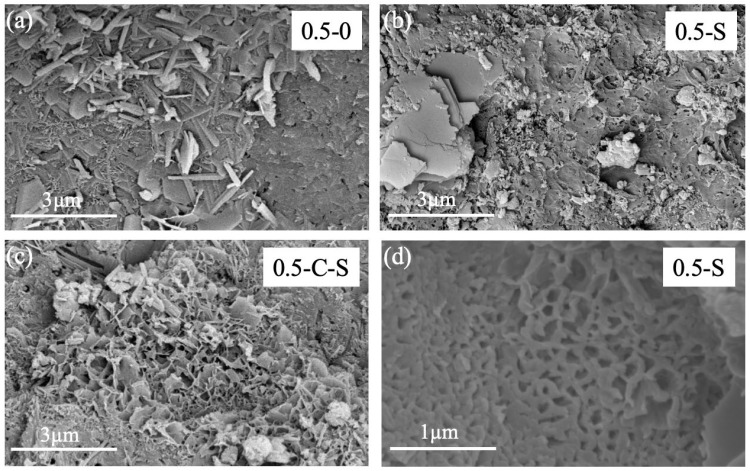
Micrographs of treated and untreated samples with w/c = 0.5: (**a**) control; (**b**) silica soaking; (**c**) carbonation and silica soaking; (**d**) silica soaking (large scale).

The accelerated carbonation modification process for LS-RCA embodies a sophisticated mechanism that revolves around the fundamental transformation of both the surface and internal structure of LS-RCA through a series of chemical reactions under tightly controlled conditions. This innovative approach is designed to significantly elevate the overall properties of LS-RCA, thereby augmenting the performance of recycled aggregate concrete in various applications. At the heart of this process lies the exposure of LS-RCA, particularly those attached with hardened cement mortar on their surfaces, to elevated concentrations of CO_2_ under potentially enhanced pressures. This environment acts as a catalyst, fostering rapid reactions between the prevalent Ca(OH)_2_ and C-S-H (calcium silicate hydrate) within the cement matrix and the abundant CO_2_. These reactions culminate in the formation of CaCO_3_ crystals and silica gel, both of which contribute to the strengthening of RCA [29]. The core strengthening mechanisms underpinning this process can be delineated as follows:

Firstly, there is the activation of the surface and interfaces. Upon initial exposure to CO_2_-rich conditions, LS-RCA undergoes a swift surface reaction, wherein Ca(OH)_2_, abundant in the hydrated cement paste, rapidly reacts with CO_2_ to produce CaCO_3_ (calcium carbonate) [30,31]. This reaction propagates rapidly as CO_2_ diffuses into the intricate microstructure of the hardened cement matrix. Secondly, there is the filling of microcracks and pores. The resultant CaCO_3_ crystals, being less soluble and more stable than their precursors, accumulate within microcracks and pores, effectively sealing them off [32]. Furthermore, the concurrent formation of silica gel, a byproduct of the reaction between C-S-H and CO_2_, reinforces this filling effect [33]. Both of these mechanisms conspire to substantially reduce the porosity and suction capacity of LS-RCA. Thirdly, there is structural densification. As microcracks and pores are progressively filled, the overall structural integrity of LS-RCA is enhanced, leading to densification. This structural transformation translates into a notable increase in compressive strength and resistance to deformation, making LS-RCA more suitable for use in structural applications. Lastly, there is the enhancement of mechanical properties. The aforementioned transformations, coupled with the reduction in porosity and improved surface-to-matrix bonding, conspire to elevate RCA’s mechanical properties [31]. Experimental studies have corroborated these findings, demonstrating improvements in LS-RCA’s crushing index by approximately 26%. Moreover, beyond mere mechanical properties, accelerated carbonation treatment also positively impacts LS-RCA’s durability characteristics. The dense, crack-free microstructure (see Figure 4 and Figure 5) fostered by this process imparts resilience against aggressive environmental factors, enhancing LS-RCA’s impermeability, frost resistance, and corrosion resistance. Specifically, water absorption is reduced by up to 31%, highlighting the substantial improvements in durability.

**Figure 6:**
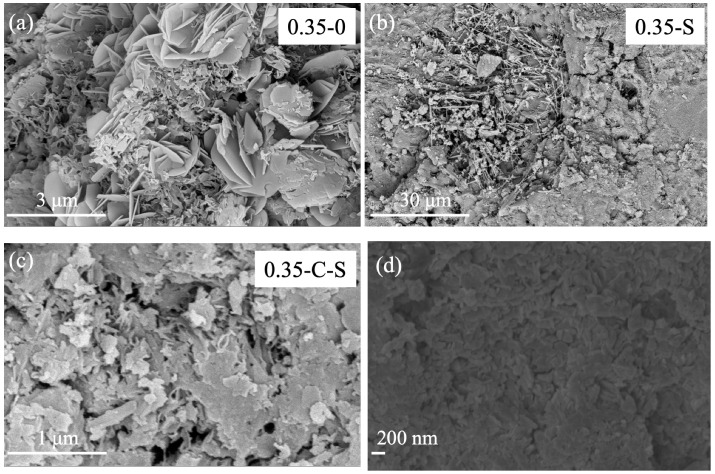
Micrographs of treated and untreated samples with w/c = 0.35: (**a**) control; (**b**) silica soaking; (**c**) carbonation and silica soaking; (**d**) silica soaking (large scale).

Contrary to the accelerated carbonation approach, the immersion of nano-silica emerges as another pivotal technique in enhancing the LS-RCA, its modification mechanism intricately weaving together complex physical and chemical interactions that yield marked improvements in LS-RCA’s properties. This multifaceted process stems fundamentally from the exceptional attributes of nano-silica particles and their capacity to intimately interact with the LS-RCA microstructurally. Firstly, the nanoscale filling effect underpins a critical aspect of this modification. Nano-silica particles, characterized by their diminutive size (in this instance, with an average diameter of 50 nanometers), boast a remarkable surface-to-volume ratio. This property endows them with the capability to effectively penetrate and occupy the myriad of microcracks, pores, and voids inherent in LS-RCA (see Figure 5 and Figure 6), often exacerbated by the crushing and recycling processes [34]. By sealing these microstructural imperfections, nano-silica significantly diminishes RA’s porosity, thereby augmenting its density and mechanical robustness. This nanoscale filling effect constitutes a cornerstone in bolstering LS-RCA’s overall structural integrity.

Secondly, the pozzolanic reaction plays a pivotal role in the modification process. Nano-silica exhibits pronounced pozzolanic activity, manifesting as its propensity to chemically interact with calcium hydroxide (Ca(OH)_2_), a prevalent component in the hydrated cement paste of RA [35]. This reactive encounter culminates in the formation of calcium silicate hydrates (C-S-H), a pivotal binder phase essential to the strength and durability of cementitious materials. The generation of additional C-S-H not only fortifies the LS-RCA matrix but also contributes to the further occlusion of microvoids and microcracks, accelerating the densification process. This pozzolanic reaction thus complements the nanoscale filling effect, fostering a synergistic enhancement of RA’s properties [36]. In essence, the nano-silica modification mechanism of LS-RCA encapsulates a harmonious interplay between nanoscale filling and pozzolanic reactivity. These mechanisms operate in tandem, each reinforcing the other, to elevate LS-RCA’s performance, rendering it a highly promising strategy for reinforcing structures.

The modification mechanisms between accelerated carbonation-modified LS-RCA (AC-LS-RCA) and nano-silica-modified LS-RCA (NS-LS-RCA) reveal profound differences, as elucidated in the following discourse. The AC-LS-RCA modification mechanism is fundamentally rooted in the chemical transformation and subsequent structural compaction. At its core lies the carbonation reaction, wherein the Ca(OH)_2_ and C-S-H (calcium silicate hydrate) components present within the adhered hardened cement paste on the AC-LS-RCA surface undergo a transformational interaction with CO_2_. This chemical interplay prompts the precipitation of CaCO_3_ crystals and silica gel, which act as effective fillers, plugging microcracks and pores within the LS-RCA matrix. This, in essence, fosters structural densification, as the accumulation of CaCO_3_ precipitates and silica gel consolidates the LS-RCA matrix, diminishing porosity and enhancing its overall compactness. Consequently, this structural consolidation diminishes water absorption and fortifies the aggregate’s mechanical strength.

Conversely, the NS-LS-RCA modification mechanism primarily harnesses the high pozzolanic effect, nucleation effect, and filling effect inherent to nano-silica. Nano-silica’s exceptional pozzolanic reactivity enables it to engage in reactions with alkaline constituents, notably Ca(OH)_2_, present in LS-RCA. These reactions stimulate the generation of additional cementitious hydration products, which not only contribute to pore filling but also refine the microstructural architecture. Furthermore, nano-silica particles serve as nucleation sites, catalyzing the crystallization of hydration products and expediting their formation, thereby enhancing the degree of hydration. Simultaneously, the nanoparticles themselves physically occupy pores, further augmenting the density and homogeneity of the LS-RCA’s microstructure.

In summary, AC-LS-RCA emphasizes chemical transformation via carbonation, leading to structural compaction through the precipitation of CaCO_3_ and silica gel. Conversely, NS-LS-RCA leverages the multifaceted attributes of nano-silica, including its pozzolanic activity, nucleation effects, and filling capabilities, to concurrently foster hydration product formation, pore filling, and interfacial bonding. These disparities underscore the distinct, yet complementary, pathways employed by AC-LS-RCA and NS-LS-RCA modifications to elevate the properties of recycled aggregates, offering insights into the nuanced mechanisms underpinning their respective enhancement strategies.

## 3. Materials and Methods

### 3.1. Materials

The OPC with a grade of 52.5 was employed as the binding material. The detailed chemical composition of this cement is presented in Table 1. To facilitate the investigation, nano-silica particles with a mean diameter of 50 nm were utilized to formulate a solution with a precise mass fraction of 2%. Additionally, commercial-grade carbon dioxide gas, boasting a purity level of 99.99%, served as the source for accelerated carbonation, enabling precise control over the carbonation process and ensuring the validity of the study.

### 3.2. Sample Preparation

To attain uniformity, LS-RCAs of sizes ranging from 4.75 to 9.5 mm underwent a rigorous crushing and sieving procedure, originating from laboratory-prepared pastes with water-cement ratios specifically tailored at 0.5 and 0.35, followed by an extended curing duration exceeding 180 days. Following this preparatory phase, the LS-RCAs were subjected to treatments as outlined in Table 2, with distinct ticks indicating the specific processes undergone. The accelerated carbonation tick denotes sole exposure to the carbonation process, whereas the nano-silica pretreatment tick signifies immersion exclusively in the nano-silica solution. The coexistence of ticks for both methods underscores a comprehensive treatment protocol, wherein samples were initially pretreated with accelerated carbonation and subsequently subjected to nano-silica immersion, thereby integrating the synergistic effects of both methodologies.

**Figure 7:**
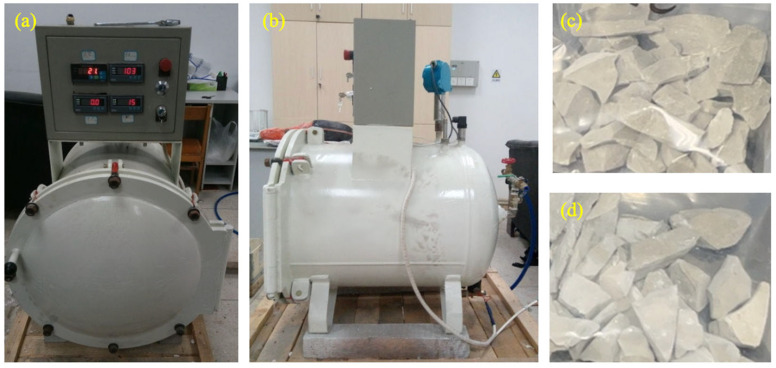
Carbonation device and the samples before and after carbonation: (**a**) front view of the carbonation device; (**b**) side view of the carbonation device; (**c**) aggregates before carbonation; (**d**) aggregates after carbonation.

For the accelerated carbonation procedure, a specialized, fully enclosed pressure vessel was employed as shown in Figure 7. Within this vessel, the prepared LS-RCAs were strategically placed on a shelf with a saturated solution of magnesium nitrate (Mg (NO_3_)_2_) positioned at the base to meticulously regulate the internal relative humidity (RH) to an optimal range of approximately 54%, fostering an ideal environment for the carbonation process. Prior to the carbonation phase, the vessel was evacuated to a pressure of −0.6 bar, subsequently admitting carbon dioxide gas of 99.99% purity, achieving a target pressure of 1 bar. A precision pressure regulator ensured the maintenance of this pressure level throughout, guaranteeing a steady and continuous supply of CO_2_ to the vessel interior. After a precise duration of 24 h of carbonation, the samples were extracted for subsequent nano-silica treatment. In the context of nano-silica pretreatment, the aggregates were immersed in a nano-silica solution of 2% concentration for 24 h under controlled conditions of 25 °C.

### 3.3. Testing

Macro-properties of LS-RCAs mainly include the crushing value and the water absorption. The crushing value test for aggregates assesses their resistance to crushing under a gradually applied compressive load, serving as an indicator of aggregate strength and suitability for various construction applications. In this test, a sample of aggregates was subjected to a standard load of 100 kN in a compression machine, and the percentage of the weight of fines produced by crushing was calculated, with a lower crushing value indicating higher strength. The water absorption test (24 h water absorption as per GB/T 25177-2010), on the other hand, measures the ability of aggregates to absorb water, which is crucial in determining their durability and potential for volumetric changes. The test involved weighing a dried aggregate sample, immersing it in water for a specified duration, and then reweighing it after removal from water. The difference in weights before and after immersion was used to calculate the percentage of water absorbed, providing insight into the aggregate’s porosity and susceptibility to moisture-related issues.

To ascertain the microscopic properties of the LS-RCAs, a systematic methodology was adopted. Initially, the aggregates were fragmented into particles with a targeted diameter of approximately 2 mm. These fine particles were then introduced into the AutoPore V system (AutoPore IV 9510, Micromeritics Instrument Corporation, Frankfurt, Germany), an instrument capable of autonomously determining porosity and pore size distribution. This step was pivotal in gaining an initial understanding of the aggregates’ internal structure.

Prior to conducting scanning electron microscopy (SEM) (JSM-7600F, JEOL Ltd., Tokyo, Japan) analysis, a rigorous surface preparation protocol was followed. The prepared RAs underwent a surface grinding process to achieve a uniform and smooth surface, which is crucial for high-resolution imaging. To prevent any potential hydration during the subsequent analysis, the samples were immersed in anhydrous ethanol, effectively mitigating the risk of water absorption. Subsequently, the samples were dried in a controlled environment using a thermostatic oven at 60°C for 12 h, ensuring that a constant weight was achieved, indicative of complete dehydration. Following the drying procedure, the samples underwent gold sputter coating and vacuum evacuation to enhance their conductivity and reduce charging artifacts during SEM analysis. The micrographs obtained provided detailed insights into the microstructure, morphology, and potential defects within the LS-RCAs.

To quantitatively evaluate the compositional aspects, particularly the content of portlandite (CH) and calcium carbonates (Cc), a thermogravimetric analysis (TGA) (Q2000, TA Instruments) was performed. In this process, powdered samples were placed in a corundum crucible and subjected to a carefully calibrated heating program. The temperature was gradually increased at a rate of 10 °C/min, commencing at 20 °C and culminating at 1000 °C, all while maintaining a constant argon flow of 20 mL/min to ensure an inert atmosphere. The TA universal analysis software was utilized to accurately quantify the mass losses during this thermal treatment, which corresponded to the dehydration of CH and the decomposition of calcium carbonates. Furthermore, to disentangle the contribution of calcium carbonates derived from the carbonation of calcium-silicate-hydrate (C-S-H) from those originating from CH carbonation, a calculation was performed. Specifically, the percentage of calcium carbonates attributed to C-S-H carbonation was approximated by subtracting the calcium carbonate content associated with CH carbonation from the total calcium carbonate content obtained from the TGA analysis. This approach provided a specific understanding of the carbonate formation mechanisms within the LS-RCAs.

## 4. Conclusions

Based on the comprehensive analysis presented in the study, the following conclusions can be drawn:

The investigation into the composite modification approach, integrating accelerated carbonation (AC) and nano-silica immersion (NS), has demonstrated remarkable potential in enhancing the utilization of LS-RCAs in sustainable construction. RCA, a significant contributor to construction waste, has traditionally struggled to match the properties of virgin aggregates. However, the innovative combination of AC and NS has significantly improved RCA’s performance, overcoming these challenges.

The nano-silica, a potent pozzolan, effectively filled micro-cracks and voids within RCA, thereby strengthening its bonding capabilities and overall strength. Meanwhile, the accelerated carbonation pretreatment accelerated LS-RCA’s natural carbonation process, resulting in the formation of calcium carbonate which further fortified the aggregate and reduced its porosity. These synergistic effects led to substantial improvements, with a notable 23% reduction in the crushing index and up to 30% decrease in water absorption rates.

It is noteworthy that the modification efficacy was more pronounced when applied to LS-RCAs derived from common-strength concrete (w/c ratio of 0.5), compared to high-strength concrete (w/c ratio of 0.35). This disparity can be attributed to the inherently looser structural framework and higher abundance of detrimental crystal structures in LS-RCAs from common-strength concrete, which were more receptive to the modification techniques.

The combined modification not only doubled the calcium carbonate content but also reduced the proportion of calcium hydrate by approximately 30%. Additionally, it led to a 15% reduction in total porosity and a roughly 20% constriction of the average pore diameter, resulting in pore refinement that equated the performance of LS-RCA samples with a water-to-cement ratio of 0.5 to those with a ratio of 0.35. This remarkable transformation underscores the profound modification potential of the proposed approach.

In conclusion, this study underscores the efficacy of harnessing accelerated carbonation in conjunction with nano-silica as a strategic method for optimizing the utilization of RCA in concrete mixes. By bolstering LS-RCA performance metrics, this approach not only enhances the sustainability of construction practices but also contributes to the circular economy by promoting the reuse of construction waste. The findings of this research pave the way for future advancements in the field of sustainable construction materials and underscore the importance of innovative modification techniques in addressing environmental challenges.

## Figures and Tables

**Table 1:** Chemical composition of binding material (OPC 52.5) (by wt.%).

Notation	CaO	SiO_2_	Al_2_O_3_	Fe_2_O_3_	MgO	TiO_2_	Na_2_O	K_2_O	LoI
Cement	64.51	21.62	5.37	3.31	2.16	0.2	0.75	1.81	0.27

**Table 2:** Treatment method of samples.

Notation	Water/Cement	Accelerated Carbonation	Nano-Silica Pretreatment
0.5-0	0.50	-	-
0.5-S	0.50	-	√
0.5-C-S	0.50	√	√
0.35-0	0.35	-	-
0.35-S	0.35	-	√
0.35-C-S	0.35	√	√

## Data Availability

Data will be available on the request from the corresponding author.
